# Herbal Medicines Induced Anticholinergic Poisoning in Hong Kong

**DOI:** 10.3390/toxins8030080

**Published:** 2016-03-18

**Authors:** Thomas Y. K. Chan

**Affiliations:** 1 Division of Clinical Pharmacology and Drug and Poisons Information Bureau, Department of Medicine and Therapeutics, Faculty of Medicine, The Chinese University of Hong Kong, Prince of Wales Hospital, Shatin, New Territories, Hong Kong, China; tykchan@cuhk.edu.hk; Tel.: +852-2632-3907; 2 Prince of Wales Hospital Poison Treatment Centre, Hong Kong, China

**Keywords:** anticholinergic poisoning, herbal medicines, tropane alkaloids, *Flos Daturae Metelis*, Hong Kong

## Abstract

In the present review, the main objective was to report the incidence and causes of herbal medicines induced anticholinergic poisoning in Hong Kong during 1989–2012 and to emphasize the importance of pharmacovigilance, investigations and preventive measures. Relevant papers, official figures and unpublished data were obtained from Medline search, the Department of Health and the Drug and Poisons Information Bureau. In the New Territories East (where ~20% of the Hong Kong population lived), the incidence of herbal medicines induced anticholinergic poisoning during 1989–1993 was 0.09 per 100,000 population. There were no confirmed cases during 1994–1996. In the whole of Hong Kong, the incidence during 2000–June 2005 was 0.03 per 100,000 population. Contamination of *Rhizoma Atractylodis* (50%) and erroneous substitution (42%) were the main causes. The incidence during 2008–2012 was 0.06 per 100,000 population. Contamination of non-toxic herbs (50%) and erroneous substitution (41%) were the main causes. In Hong Kong, contamination of non-toxic herbs by tropane alkaloids and substitution of *Flos Campsis* by toxic *Flos Daturae Metelis* were the predominant causes of herbal medicines induced anticholinergic poisoning. Systematic studies along the supply chain are necessary to identify the likely sources of contamination. If erroneous substitution of *Flos Campsis* by *Flos Daturae Metelis* could be prevented, 40% of herbal medicines induced anticholinergic poisoning would not have occurred. Regular inspection of the retailer, continuing education for the staff in the herbal trade and repeated publicity measures will also be required. Pharmacovigilance of herbal medicines should help determine the incidence and causes of adverse reactions and monitor the effectiveness of preventive measures.

## 1. Introduction

In Hong Kong, both traditional medicine and western medicine are widely accepted by the public. In the 2011–2012 Thematic Household Survey of persons aged ≥15 years, 8.2% of individuals with chronic health conditions took herbal medicines, 48.9% took drugs, and 9.3% took both herbal medicines and drugs regularly in the 6 months before enumeration [1]. Adverse reactions to herbal medicines and drugs accounted for 0.2% and 4.4% of acute admissions to general medical wards [2,3]. Surveillance of acute hospital admissions since 1989 [4] and spontaneous reports received since 2007 by the four territory-wide poison control units [5,6,7] of the Hong Kong Poison Control Network (http://www.hkpcn.org.hk) indicate that herbs containing or contaminated with *Aconitum* alkaloids (e.g., aconitine, mesaconitine, hypaconitine, yunaconitine and crassicauline A) and tropane alkaloids (e.g., atropine, hyoscyamine and scopolamine) remain the two most important causes of herbal poisoning. The clinical presentations and investigations of *Aconitum* alkaloid poisoning caused by aconite roots or contaminated herbs during1989–2011 have recently been reported [8,9].

The tropane alkaloids compete with acetylcholine for binding sites on the muscarinic receptors, causing the classical anticholinergic toxidrome (antimuscarinic effects) [10,11,12]. The peripheral effects of muscarinic receptor antagonism include: mydriasis, blurred vision, dry mouth, flushed dry skin, dry mucous membranes, hyperthermia, sinus tachycardia, hypertension, decreased bowel motility, and urinary retention. The central effects of muscarinic receptor antagonism include hallucinations, delirium, drowsiness, slurred speech, agitation, amnesia, ataxia, myoclonus, seizures, and coma. The severity of anticholinergic toxicity is generally dose-dependent. The tropane alkaloid content is the highest in the flowers of the *Datura* species [12]. Thus, overdose of their flowers as herbal medicines or consumption of non-toxic herbs contaminated by such plant parts is associated with a higher risk of anticholinergic poisoning [10]. Milder anticholinergic toxicity may only require supportive care. In severe cases (e.g., profound delirium), physostigmine, a cholinesterase inhibitor, should be used to reverse anticholinergic toxicity [12].

In the present review, the main objective is to report the incidence and causes of herbal medicines induced anticholinergic poisoning in Hong Kong during 1989–2012 and to emphasize the importance of pharmacovigilance, investigations and preventive measures.

## 2. Methodology

To identify journal articles on herbal medicines related anticholinergic poisoning, a search of Medline (1989–21 December 2015) was performed, using herbal medicines, anticholinergics, atropine, hyoscyamine, scopolamine, anticholinergic poisoning and *Datura* as the keywords. The official figures and publications on herbal poisoning were provided by the Department of Health (http://www.dh.gov.hk/eindex.html). Additional articles and unpublished data were provided by the Drug and Poisons Information Bureau [4].

To calculate the incidence of herbal medicines induced anticholinergic poisoning, the mid-year population of Hong Kong or the population of the New Territories East (NTE) (*i.e.*, the catchment areas of the Prince of Wales Hospital, PWH) was used, where appropriate. Data on the population size were provided by the Census and Statistics Department. The overall incidence was calculated from the number of cases and the population size in aggregate during that period.

Herbal poisoning was diagnosed using well-established criteria [4,6]. In brief, the adverse reactions, which occurred only after exposure to herbal medicines, could not be reasonably explained by the underlying medical conditions or the effects of concomitant drugs, if any. In the case of anticholinergic poisoning, the characteristic clinical features (e.g., acute confusion, mydriasis, dry mouth and sinus tachycardia) [10,11,12] should be present. A multidisciplinary team reviewed the history, clinical features, prescriptions, herbal remnants and toxicological laboratory findings [6]. The causes of anticholinergic poisoning could then be categorized as: overdoses (larger than the recommended doses used), erroneous substitution (non-toxic *Flos Campsis* or, rarely, *Flos Rhododendri mollis* substituted by *Flos Daturae Metelis* due to their very similar appearances) and contamination (non-toxic herbs contaminated by plants, herbs or foreign matters containing tropane alkaloids) [13,14,15,16]. *Flos Daturae Metelis* contains 0.3%–0.43% alkaloids (scopolamine 85%, hyoscyamine/atropine 15%) [10].

As this review was based on published data and information freely available in the public domain, research ethics committee approval was not required.

## 3. Results

There were four case series of herbal medicines induced anticholinergic poisoning reported by the poison control units in Hong Kong, covering the periods from 1989–1993 [5], 2000–June 2005 [13,14] to 2008–2012 [15]. These four reports would be reviewed in detail (Table 1). A paper with only the hospital data from June 2004 to March 2012 [16] was excluded because most of these cases were already included in the three official reports [13,14,15].

The first case series was from the NTE [5], where ~20% of the Hong Kong population lived and the PWH was, until December 1996, the only general hospital in the region. During 1989–1993, there were five cases and the incidence of herbal medicines induced anticholinergic poisoning was 0.09 per 100,000 population (Table 1). Prescriptions were available for review in three cases and the causes of herbal poisoning were contamination (40%) and substitution (20%).

The Drug and Poisons Information Bureau provided on-site consultations to the PWH and drug and poisons information service to the whole of Hong Kong from 1988 to 2001. According to its database on all causes of herbal poisoning, there were no confirmed cases of anticholinergic poisoning in the NTE during 1994–1996 (Table 1). As from 1997 to 1999, complete data were not available because two new general hospitals in the NTE region were open in 1997–1998 and the Department of Health was establishing a territory-wide pharmacovigilance system for herbal medicines.

Three case series were based on the confirmed reports received by the Department of Health from the whole of Hong Kong (Table 1). From 2000 to June 2005, there were 12 cases of anticholinergic poisoning, with an overall incidence of 0.03 per 100,000 population [13,14]. The causes of poisoning were contamination of *Rhizoma Atractylodis* (50%), substitution (42%) and overdoses (8%).

During 2008–2012, there were 22 cases of anticholinergic poisoning, with an overall incidence of 0.06 per 100,000 population [15]. The causes of poisoning included contamination (50%), substitution (41%) and overdoses (5%). Of the 11 cases of contamination, the herb involved was identified in 6 (*Rhizoma Atractylodis* in 3, *Radix Aucklandiae* in 2 and *Radix Strobilanthis Forrestii* in 1).

## 4. Discussion

Given the increasing use of herbal medicines globally and the need for knowing the causes of adverse reactions, the monitoring of the safety and quality herbal medicines within the national pharmacovigilance system should be enhanced [17]. In Hong Kong, pharmacovigilance has long been broadened to cover herbal medicines [4,18]. The territory-wide spontaneous reporting system run by the Department of Health [13,14] is strengthened by the close networking of the poison control units through the establishment of the Hong Kong Poison Control Network in April 2007. Poisoning cases with potential public health impact (including herbal medicines related adverse reactions) are reported by fax to the Department of Health [19] and the Hospital Authority Head Office. Since the public hospitals under the Hospital Authority account for 93% of the in-patient service in Hong Kong, most of the hospitalized cases should have been identified. As the surveillance and investigations of poisoning incidents are enhanced, control measures can be implemented in a timely manner [9,18]. Poisoning incidents requiring urgent attention include adverse reactions due to contamination or substitution by toxic herbs.

Like aconite poisoning [8,9,20], anticholinergic poisoning is a high priority, in view of the potency of tropane alkaloids, the severity of toxic symptoms [12] and the risk of death [21]. Because tropane alkaloids are fairly heat-stable [22], boiling (decoction) of herbs does not provide protection. The incidence and the underlying reasons for the continuing occurrence of herbal medicines induced anticholinergic poisoning should be identified.

This review systematically analyzed the best available data on the incidence and causes of herbal medicines related anticholinergic poisoning in an Asian community where both herbal and western medicines are popular (Table 1). In the NTE, the incidence of anticholinergic poisoning decreased from 0.09 in 1989–1993 to 0 in 1994–1996 per 100,000 population. A marked decrease in the incidence of aconite poisoning was also seen in the NTE, from 0.60 in 1989–1991 to 0.16 in 1992–1993 and 0.17 in 1996–1998 per 100,000 population) [8]. Perhaps, the publicity measures in late 1991 by the health authority to warn the public, healthcare professionals and herbalists of the toxicity of aconite roots [23] helped promote the appropriate use of other herbs as well. In the whole of Hong Kong, the number of reports received by the Department of Health indicated an increase in the incidence of anticholinergic poisoning from 0.03 in 2000–June 2005 to 0.06 in 2008–2012 per 100,000 population. As anticholinergic poisoning is a serious condition with characteristic clinical features [10,11,12] and there is close networking of poison control units [6,19] with well-established channels for reporting by the healthcare professionals [13,14], under-reporting and missed diagnosis are less likely.

There are a variety of reasons why adverse reactions to herbal medicines occur [6,24], but their relative importance is rarely studied in a systematic manner [8,9]. In anticholinergic poisoning, the available information from Hong Kong clearly indicated that contamination of other non-toxic herbs by plants, herbs or foreign matters containing tropane alkaloids and erroneous substitution of *Flos Campsis* by toxic *Flos Daturae Metelis* were always the two predominant causes (Table 1). Contamination of non-toxic herbs by (impurities containing) tropane alkaloids should be suspected if anticholinergic poisoning occurred even though *Flos Daturae Metelis* and related herbs were not prescribed or dispensed. Contamination of dispensed herbs could occur at various stages, such as after harvest, processing, transportation, packing, storage and dispensing. On-site investigations by the Department of Health would exclude accidental mix-up and cross-contamination at the retailer. Samples of dispensed herbs were examined for gross contamination and analyzed for the presence of tropane alkaloids. If contamination was confirmed, the public, the healthcare professionals and the herbal trade should be warned. If herbal samples from the wholesaler were also contaminated and contamination must have occurred before import into Hong Kong, the regulatory authorities of exporting countries should also be notified for follow-up actions. So far, *Rhizoma Atractylodis* was involved more often than other non-toxic herbs (see Results). Further systematic studies along the supply chain are required to identify the likely sources of contamination so that risk management strategies can be formulated.

On-site inspection by the Department of Health might reveal mislabeling of *Flos Daturae Metelis* as *Flos Campsis* in the involved herbal shops [15]. The dried forms of *Flos Campsis* and *Flos Daturae Metelis* have very similar appearances. Consequently, when *Flos Campsis* was prescribed, *Flos Daturae Metelis* was actually dispensed. The recommended doses for *Flos Daturae Metelis* and *Flos Campsis* are 0.3–0.6 g and 5–9 g, respectively [15]. Anticholinergic poisoning invariably occurred because of the overdose (8–15 times) since *Flos Daturae Metelis* was dispensed according to the prescribed dose for *Flos Campsis*. Therefore, regular inspection of the retailer, continuing education for the staff in the herbal trade and repeated publicity measures would be required. If erroneous substitution of *Flos Campsis* by *Flos Daturae Metelis* could be prevented, the recent data suggested that 40% of the anticholinergic poisoning would not have occurred (Table 1).

## 5. Conclusions

In Hong Kong, the incidence of herbal medicines induced anticholinergic poisoning increased between 2000–June 2005 and 2008–2012 from 0.03 to 0.06 per 100,000 population. The most important causes were contamination of non-toxic herbs by tropane alkaloids and substitution of *Flos Campsis* by toxic *Flos Daturae Metelis*. Systematic studies along the supply chain are necessary to identify the likely sources of contamination. Regular inspection of the retailer, continuing education for the staff in the herbal trade and repeated publicity measures will also be required. Pharmacovigilance of herbal medicines should help determine the incidence and causes of adverse reactions and monitor the effectiveness of preventive measures.

## Figures and Tables

**Table 1:** Incidence (per 100,000 population) and causes of herbal medicines induced anticholinergic poisoning.

Study Details	1989–1993 ^a^	1994–1996 ^b^	2000–June/2005 ^c,d^	2008–2012 ^e^
Number of subjects	5	0	12	22
Males:females	0:5		-	7:15
Median (range) age (y)	33 (27–39)		-	50.5 (4–79)
Total population ('000)	5260.1 ^f^	3938.7 ^f^	37044.3 ^g^	35181.0 ^g^
Overall incidence	0.09	0	0.03	0.06
Causes (% of cases)				
Overdoses ^h^	0		8	5 ^h^
Contamination ^i^	40		50	50
Erroneous substitution	20 ^j^		42 ^k^	41 ^k^
Undetermined	40		0	5 ^l^

^a^ Data from the New Territories East (~20% of the Hong Kong population) [5]; ^b^ Unpublished data from the New Territories East provided by the Drug and Poisons Information Bureau; ^c,d,e^ Data from the whole of Hong Kong based on confirmed reports received by the Department of Health [13,14,15]; ^f^ Catchment population of the Prince of Wales Hospital in the New Territories East; ^g^ Mid-year population of Hong Kong; ^h^ Overdoses (larger than the recommended doses used); Substitution (^i^ *Flos Rhododendri mollis* and non-toxic ^j^ *Flos Campsis* substituted by *Flos Daturae Metelis*); ^k^ Contamination (non-toxic herbs contaminated by plants, herbs or foreign matters containing tropane alkaloids); ^l^ Self-prescription of unknown herb belonging to the genus *Datura* with obvious overdose in one case.

## Abbreviations

The following abbreviations are used in this manuscript:

NTE: New Territories East
PWH: Prince of Wales Hospital
